# Supplementary material for: Synthesis, Structural Versatility, Magnetic Properties, and I− Adsorption in a Series of Cobalt(II) Metal–Organic Frameworks with a Charge-Neutral Aliphatic (O,O)-Donor Bridge
Source: Nanomaterials (Basel). 2023 Oct 16;13(20):2773. doi: 10.3390/nano13202773 (PMC10609582; doi:10.3390/nano13202773)
Supplement: Supplementary file 1 [file nanomaterials-13-02773-s001.zip › nanomaterials-2653490-supplementary.pdf]

# Synthesis, Structural Versatility, Magnetic Properties, and I⁻ Adsorption in a Series of Cobalt(II) Metal–Organic Frameworks with a Charge-Neutral Aliphatic (O,O)-Donor Bridge

**Ksenia D. Abasheeva [1,2], Pavel A. Demakov [1,*], Evgeniya V. Polyakova [1], Alexander N. Lavrov [1], Vladimir P. Fedin [1] and Danil N. Dybtsev [1,*]**

1 Nikolaev Institute of Inorganic Chemistry SB RAS, 3 Lavrentiev Ave., Novosibirsk 630090, Russia; k.abasheeva@g.nsu.ru (K.D.A.); e.polyakova.niic@gmail.com (E.V.P.); lavrov@niic.nsc.ru (A.N.L.); cluster@niic.nsc.ru (V.P.F.)

2 Department of Natural Sciences, Novosibirsk State University, 2 Pirogova St., Novosibirsk 630090, Russia

* Correspondence: demakov@niic.nsc.ru (P.A.D.); dan@niic.nsc.ru (D.N.D.)

## Supplementary Information

**Table S1.** Single-crystal X-ray diffraction and structure refinement details.

| | **1** | **2** | **3** | **3-I*** | **4*** |
|---|---|---|---|---|---|
| Chemical formula | $C_{24}H_{42}Co_3N_4O_{16}$ | $C_8H_{18}CoN_2O_8$ | $C_{30}H_{69.30}Co_2N_{14}O_{26.66}$ | $C_{30}H_{65.70}Co_2I_2N_{12}O_{18.85}$ | $C_{30}H_{68}Co_2N_{14}O_{25}$ |
| $M_r$, g/mol | 819.40 | 329.17 | 1170.75 | 1267.90 | 1142.84 |
| Crystal system | Monoclinic | Orthorhombic | Monoclinic | Monoclinic | Monoclinic |
| Space group | $P2_1/c$ | *Pnma* | $P2_1/n$ | $P2_1$ | $C2/c$ |
| Temperature, K | 100 | 140 | 140 | 100 | 140 |
| *a*, Å | 9.537(2) | 8.5236(6) | 16.5626(5) | 16.509(3) | 21.3012(7) |
| *b*, Å | 21.536(4) | 15.1349(11) | 17.1454(5) | 17.191(3) | 11.7009(8) |
| *c*, Å | 15.872(3) | 8.9094(8) | 17.1992(5) | 17.246(3) | 21.7341(7) |
| b, ° | 100.64(3) | 90 | 91.262(3) | 90.35(3) | 97.265(3) |
| *V*, Å$^3$ | 3203.9(11) | 1149.35(16) | 4882.9(2) | 4894.4(15) | 5373.6(4) |
| *Z* | 4 | 4 | 4 | 4 | 4 |
| *F*(000) | 1692 | 684 | 2458 | 2562 | 2400 |
| *D*(calc.), g·cm$^{-3}$ | 1.699 | 1.902 | 1.593 | 1.721 | 1.413 |
| μ, mm$^{-1}$ | 1.825 | 1.536 | 0.782 | 2.277 | 0.707 |
| Crystal size, mm | 0.15 × 0.12 × 0.10 | 0.63 × 0.59 × 0.26 | 0.56 × 0.49 × 0.41 | 0.08 × 0.03 × 0.03 | 0.51 × 0.13 × 0.12 |
| θ range for data collection, ° | 1 .69 < θ < 26.67 | 2.65 < θ < 25.35 | 2.10 < θ < 25.35 | 1.24 < θ < 25.41 | 2.15 < θ < 25.35 |
| No. of reflections: measured / independent / observed [$I > 2\sigma(I)$] | 13002 / 5834 / 5490 | 3340 / 1098 / 962 | 23311 / 8866 / 8410 | 27674 / 15136 / 14873 | 11197 / 4843 / 4174 |
| $R_{int}$ | 0.0464 | 0.0229 | 0.0244 | 0.0345 | 0.0274 |
| Index ranges | –11 < h < 11<br>–24 < k < 25<br>–19 < l < 15 | –8 < h < 10<br>–18 < k < 13<br>–10 < l < 6 | –16 < h < 19<br>–20 < k < 20<br>–20 < l < 20 | –19 < h < 18<br>–19 < k < 19<br>–19 < l < 19 | –18 < h < 25<br>–11 < k < 14<br>–26 < l < 26 |
| Final *R* indices [$I > 2\sigma(I)$] | $R_1$ = 0.0380<br>$wR_2$ = 0.0970 | $R_1$ = 0.0247<br>$wR_2$ = 0.0666 | $R_1$ = 0.0988<br>$wR_2$ = 0.2114 | $R_1$ = 0.1194<br>$wR_2$ = 0.2945 | $R_1$ = 0.0943<br>$wR_2$ = 0.2340 |
| Final *R* indices (all data) | $R_1$ = 0.0402<br>$wR_2$ = 0.0983 | $R_1$ = 0.0292<br>$wR_2$ = 0.0698 | $R_1$ = 0.1021<br>$wR_2$ = 0.2128 | $R_1$ = 0.1206<br>$wR_2$ = 0.2952 | $R_1$ = 0.1055<br>$wR_2$ = 0.2417 |

| | | | | | |
|---|---|---|---|---|---|
| Goodness-of-fit on $F^2$ | 1.068 | 1.064 | 1.210 | 1.078 | 1.052 |
| Largest diff. peak, hole, $e$/Å$^3$ | 1.08, –0.55 | 0.30, –0.34 | 1.24, –0.98 | 2.47, –2.55 | 1.27, –1.07 |

* In **4**, a content of directly localized guest water molecules was successfully refined as 0.7 $H_2O$ per formula unit. These species do not fill the voids in coordination framework completely. After successful localization of all the possible nitrates and water molecules, PLATON SQUEEZE [MS57] procedure was implemented to analyze the non-ordered electron density in the residual voids. The corresponding electron count (91 $e^-$ in 187 Å$^3$ per unit cell or 22.75 $e^-$ in *ca*. 47 Å$^3$ per formula unit) was reasonably attributed to additional 2.3 $H_2O$ molecules (23 $e^-$) per f.u., resulWting in $[Co_2(DMF)_2(odabco)_4](NO_3)_4{\cdot}3H_2O$ as a final formula for **4** crystal. For **3-I**, 1.5 I atoms and 1.5 $H_2O$ per f.u. were localized directly. Similar SQUEEZE procedure gave non-ordered electron count of 250 $e^-$ in 440 Å$^3$ per unit cell in the residual voids (62.5 $e^-$ in 110 Å$^3$ per f.u.), which was reasonably attributed to additional $0.5I^-{+}1.0NO_3^-{+}0.35H_2O$ (62.5 $e^-$) of non-ordered guest species, accordingly to the necessary electroneutrality of the crystal and the elemental analysis data providing some experimental restrictions on the iodine content. Thus, $[Co_2(H_2O)(NO_3)(odabco)_5]I_2(NO_3){\cdot}1.85H_2O$ was found as a final formula of **3-I** crystal.

**Table S2.** Bond lengths (in Å) in the metal coordination environment for **1–4**.

| | **1** | **2** | **3** | **3-I** | **4** |
|---|---|---|---|---|---|
| Co–O(μ-$\kappa^1$,$\kappa^1$-COO) | 1.999(2)<br>…<br>2.107(2) | 2.1282(13)<br>…<br>2.1573(14) | | | |
| Co–O($\kappa^1$-COO) | 2.004(2)<br>…<br>2.012(2) | | | | |
| Co–O(odabco) | 1.9700(19)<br>…<br>2.0106(19) | | 1.971(5)<br>…<br>2.127(5) | 1.936(16)<br>…<br>2.160(18) | 1.940(4)<br>…<br>2.122(4) |
| Co–O($H_2O$) | | 2.0403(15) | 1.998(5) | 1.969(17);<br>2.010(16) | |
| Co–O($NO_3$) | | | 2.214(5);<br>2.360(5) | 2.169(16)<br>…<br>2.343(17) | |
| Co–O(DMF) | | | | | 2.147(5) |

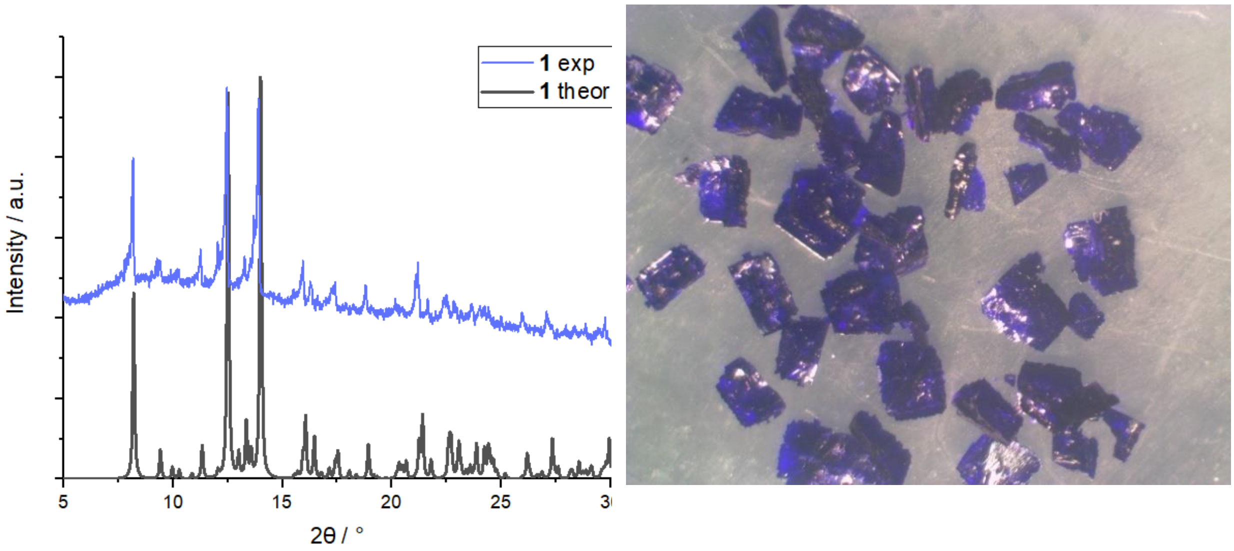


(a) (b)

**Figure S1.** Experimental and theoretical PXRD patterns of **1** (**a**). Digital photograph of the freshly filtered sample of **1** (**b**).

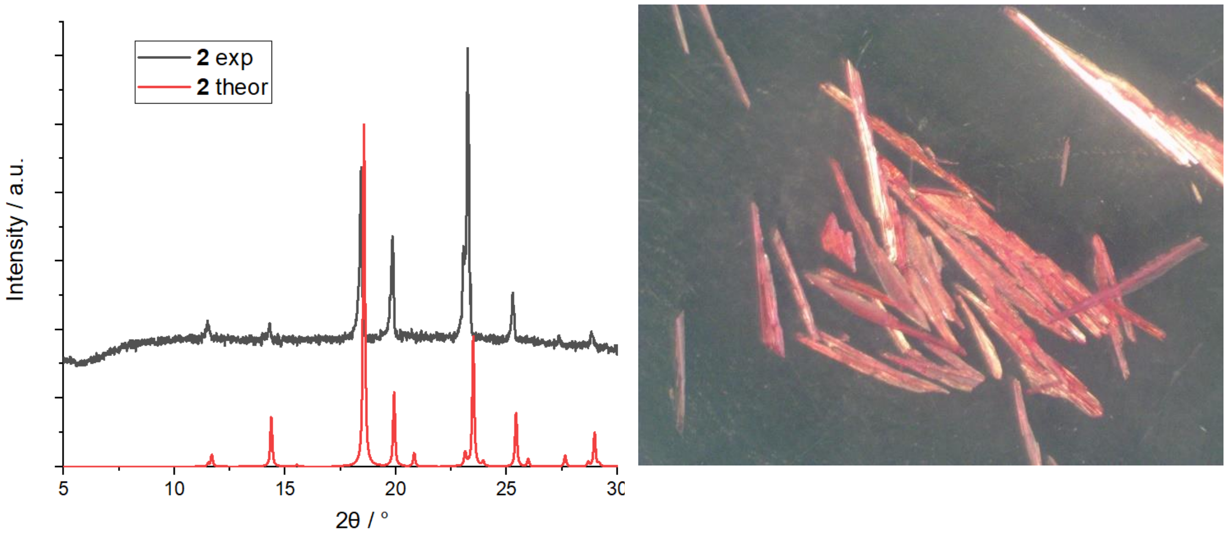


(**a**) (**b**)

**Figure S2.** Experimental and theoretical PXRD patterns of **2** (**a**). Digital photograph of the freshly filtered sample of **2** (**b**).

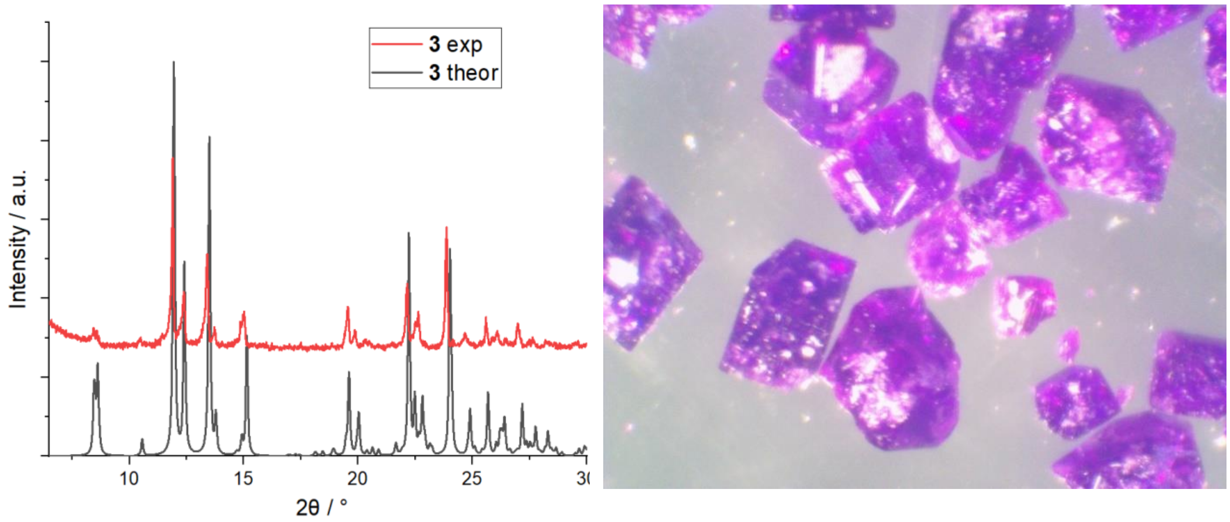


(**a**) (**b**)

**Figure S3.** Experimental and theoretical PXRD patterns of **3** (**a**). Digital photograph of the freshly filtered sample of **3** (**b**).

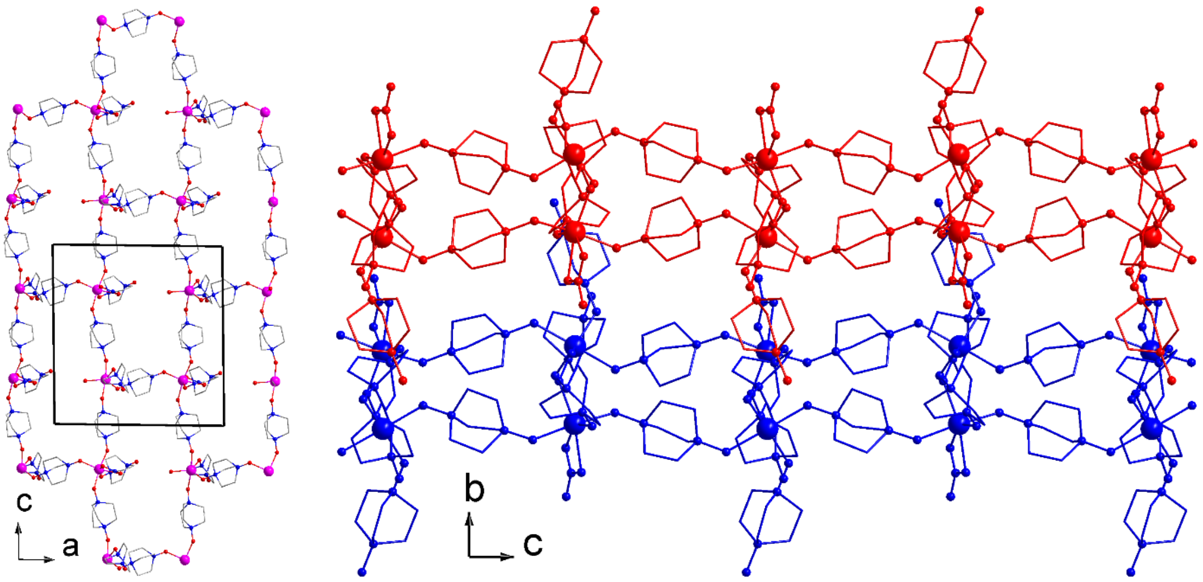


(a) (b)

**Figure S4.** Fragment of coordination layer in **3**, viewed along the *b* axis (**a**). Packing of two closest layers in **3**, viewed along the *a* axis (**b**)

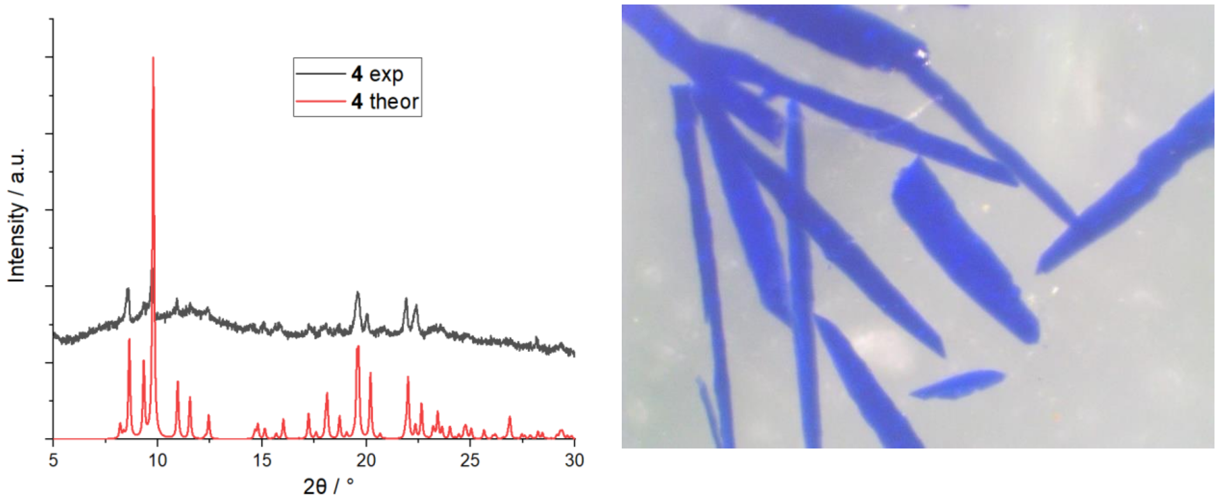


(a) (b)

**Figure S5.** Experimental and theoretical PXRD patterns of **4** (**a**). Digital photograph of the freshly filtered sample of **4** (**b**).

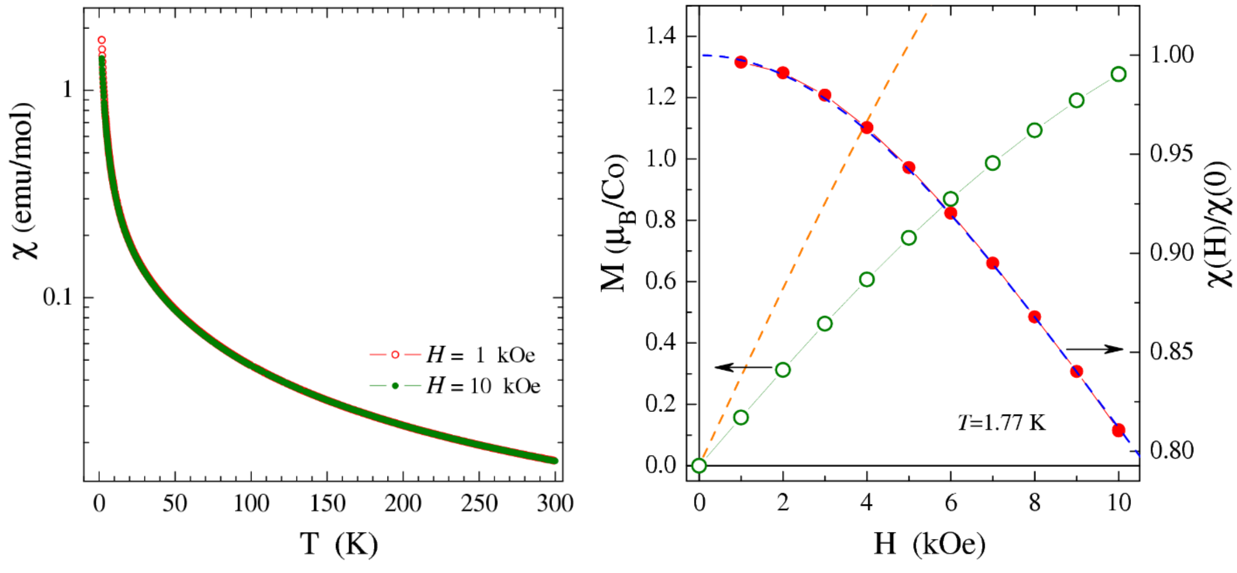


(**a**) (**b**)

**Figure S6.** (**a**) Temperature dependences of the magnetic susceptibility χ measured for **3** at magnetic fields H = 1; 10 kOe. (**b**) Magnetic field dependences of the magnetization M per one Co ion (open green circles) and normalized magnetic susceptibility χ(H)/χ(0) (solid red circles) measured for **3** at T = 1.77 K. The dashed blue line shows the best fit to the χ(H)/χ(0) data of the Brillouin function $B_S(g\mu_B SH/k_B T)$ with the magnetic moments S = 3/2 and the g-factor g = 2.48; the corresponding magnetization $M_f = g\mu_B SB_S$ is depicted by the dashed orange line.

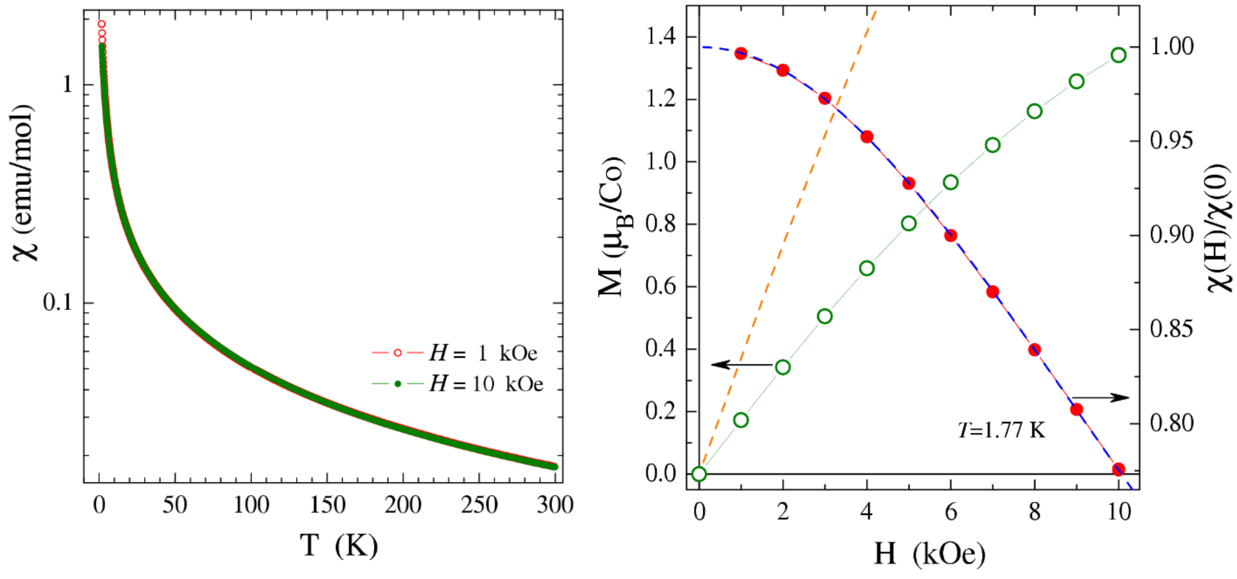


(**a**) (**b**)

**Figure S7.** (**a**) Temperature dependences of the magnetic susceptibility χ measured for **4** at magnetic fields H = 1; 10 kOe. (**b**) Magnetic field dependences of the magnetization M per one Co ion (open green circles) and normalized magnetic susceptibility χ(H)/χ(0) (solid red circles) measured for **4** at T = 1.77 K. The dashed blue

line shows the best fit to the $\chi(H)/\chi(0)$ data of the Brillouin function $B_S(g\mu_B SH/k_B T)$ with the magnetic moments S = 3/2 and the g-factor g = 2.80; the corresponding magnetization $M_f = g\mu_B S B_S$ is depicted by the dashed orange line.

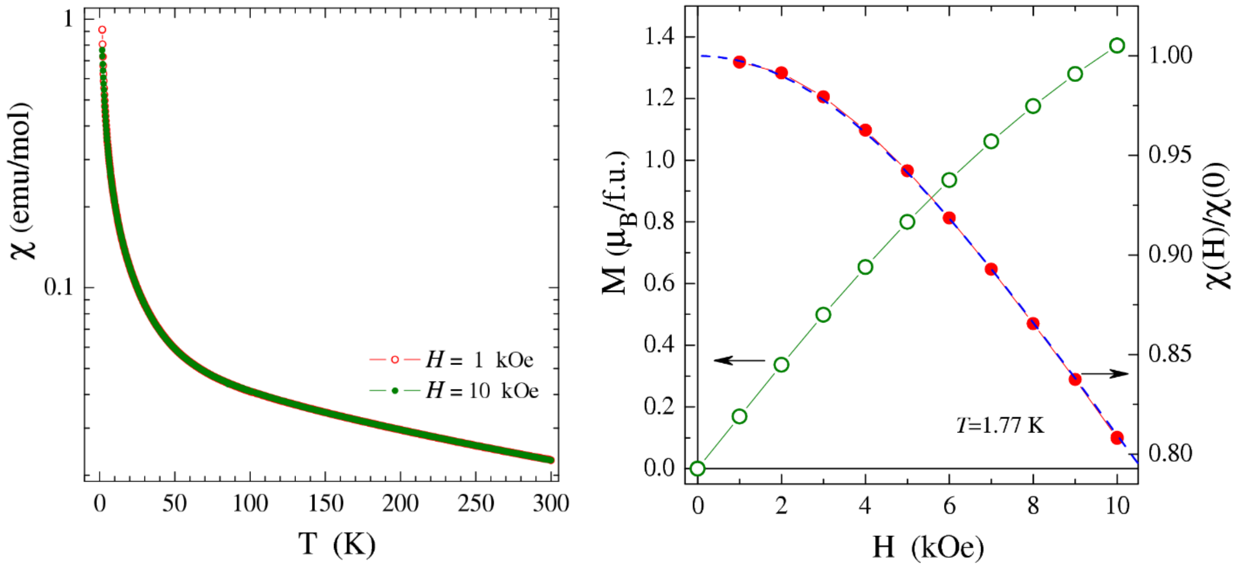


(**a**) (**b**)

**Figure S8.** (**a**) Temperature dependences of the magnetic susceptibility χ measured for **1** at magnetic fields H = 1; 10 kOe. (**b**) Magnetic field dependences of the magnetization M per formula unit (open green circles) and normalized magnetic susceptibility $\chi(H)/\chi(0)$ (solid red circles) measured for **1** at T = 1.77 K. The dashed blue line shows the best fit to the $\chi(H)/\chi(0)$ data of the Brillouin function $B_S(g\mu_B SH/k_B T)$ with the magnetic moments S = 3/2 and g = 2.50.

**Table S3.** $I^-/NO_3^-$ ratio and derived extent of the nitrate substitution by iodide after immersion of **3** in NaI solutions

| № of solution | NaI concentration, M | Immersion time, days | $I^-/NO_3^-$ molar ratio | Substitution degree, % |
|---|---|---|---|---|
| 1 | $1.0 \cdot 10^{-1}$ | 7 | 1.3 | 75 |
| | | 1 | 0.15 | 17 |
| 2 | $3.2 \cdot 10^{-2}$ | 7 | 0.91 | 64 |
| | | 1 | 0.21 | 23 |
| 3 | $1.0 \cdot 10^{-2}$ | 7 | 0.91 | 64 |
| | | 1 | 0.16 | 18 |
| 4 | $3.2 \cdot 10^{-3}$ | 1 | 0.10 | 12 |
| 5 | $1.0 \cdot 10^{-3}$ | 1 | $9.1 \cdot 10^{-2}$ | 11 |
| 6 | $3.2 \cdot 10^{-4}$ | 1 | $2.4 \cdot 10^{-2}$ | 3.2 |
| 7 | $1.0 \cdot 10^{-4}$ | 1 | $7{,}7 \cdot 10^{-3}$ | 1.0 |

**Table S4.** $I^-/NO_3^-$ ratio and derived extent of the nitrate substitution by iodide after step-by-step immersion of **3** in NaI and $NaNO_3$ solutions

| № of step | Solution concentration | Immersion time, days | $I^-/NO_3^-$ molar ratio | Substitution degree, % | 3 recyclization degree, % |
|---|---|---|---|---|---|
| 1 | $1.0 \cdot 10^{-1}$M NaI | 1 | 0.15 | 17 | 89 |
| 2 | $1.0 \cdot 10^{-1}$M $NaNO_3$ | 1 | 0.015 | 2.0 | |

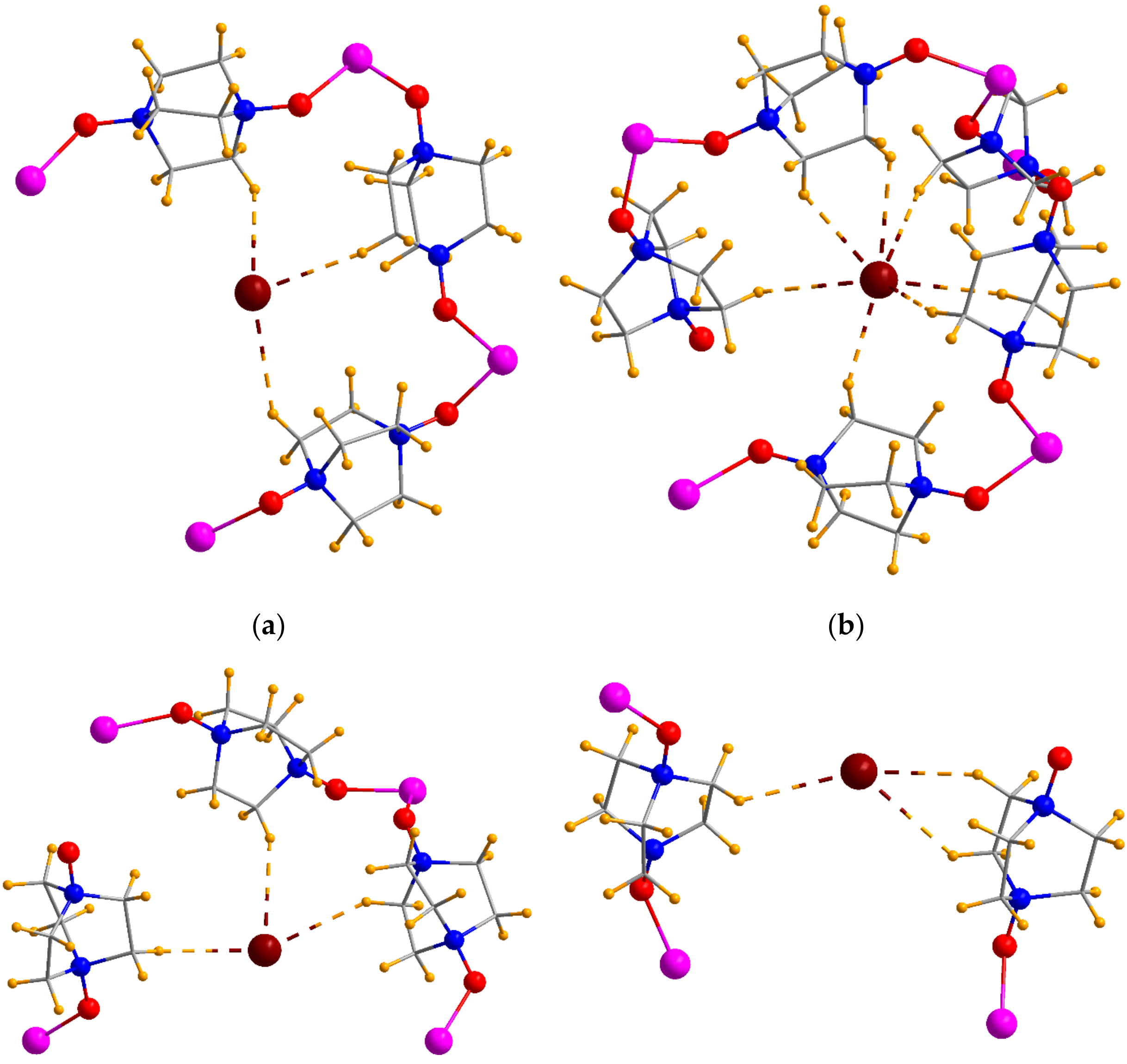

(**a**) (**b**) (**c**) (**d**)

**Figure S9.** Iodide molecular environment in **3-I:** for I3 (**a**), I4 (**b**), I5 (**c**) and I6 (**d**). H atoms are shown orange. Dashed lines present I...H contacts shorter than 3.2 Å.

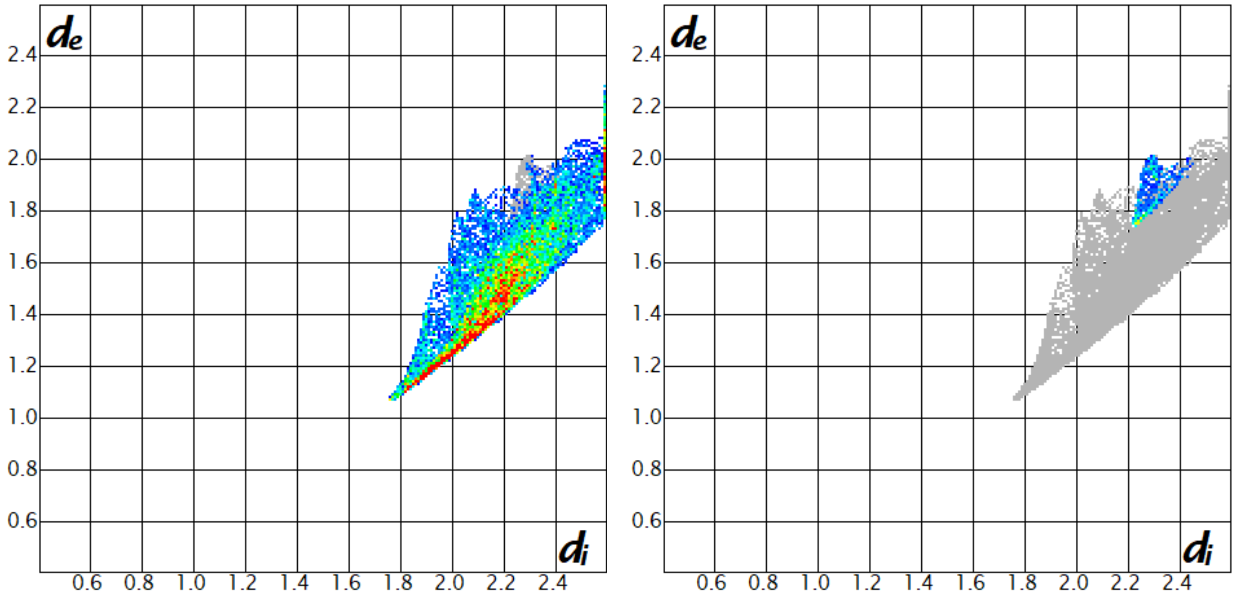


(**a**) (**b**)

**Figure S10.** I…H (**a**) and I…O (**b**) contact fingerprints for the I1 atom in the crystal structure of **3-I**.

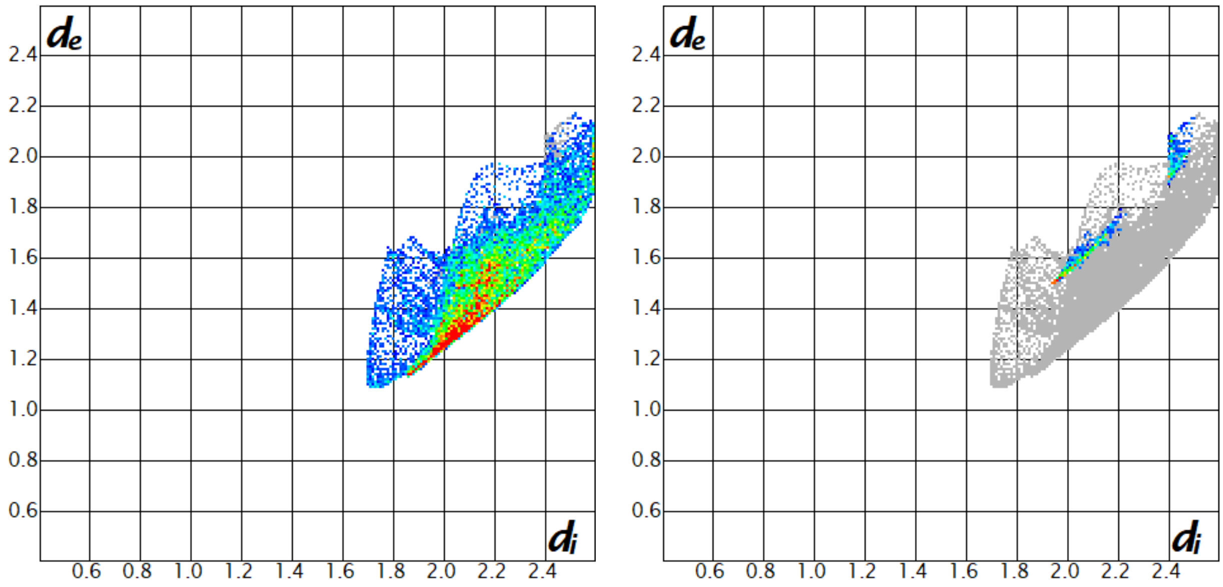


(**a**) (**b**)

**Figure S11.** I…H (**a**) and I…O (**b**) contact fingerprints for the I2 atom in the crystal structure of **3-I**.

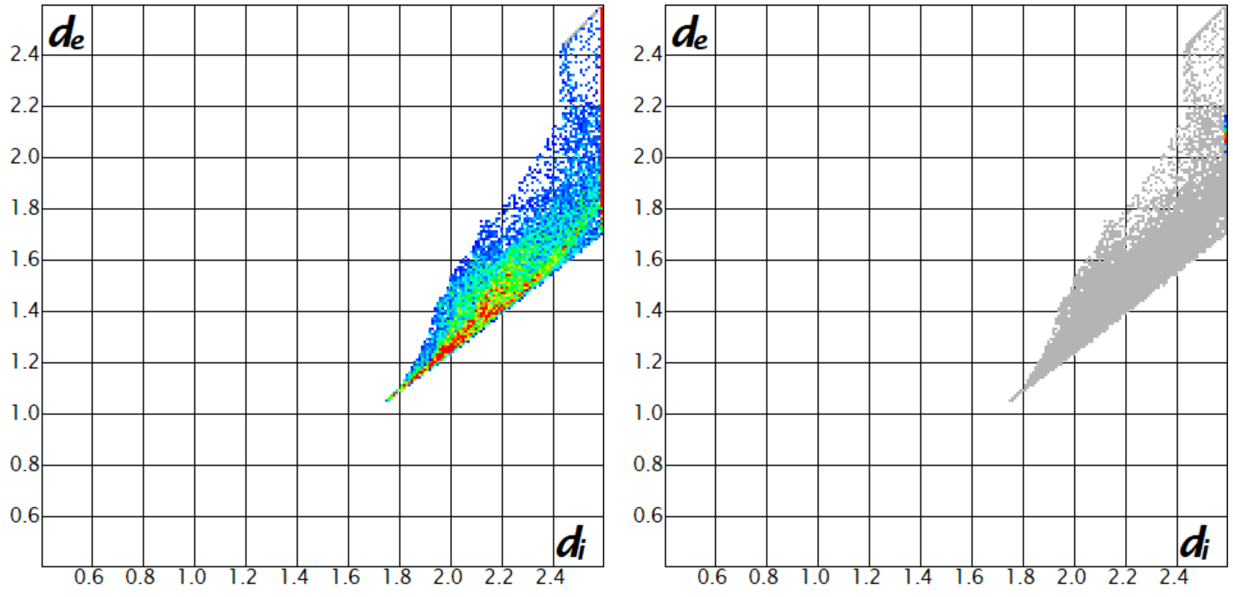


(**a**) (**b**)

**Figure S12.** I…H (**a**) and I…O (**b**) contact fingerprints for the I3 atom in the crystal structure of **3-I**.

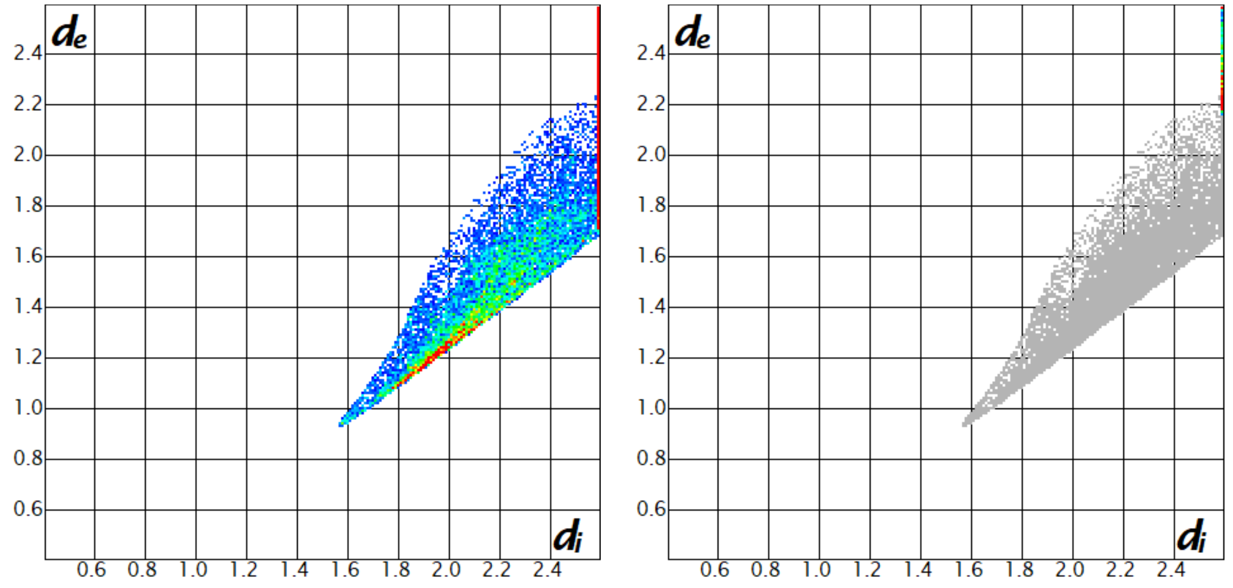


(**a**) (**b**)

**Figure S13.** I…H (**a**) and I…O (**b**) contact fingerprints for the I4 atom in the crystal structure of **3-I**.

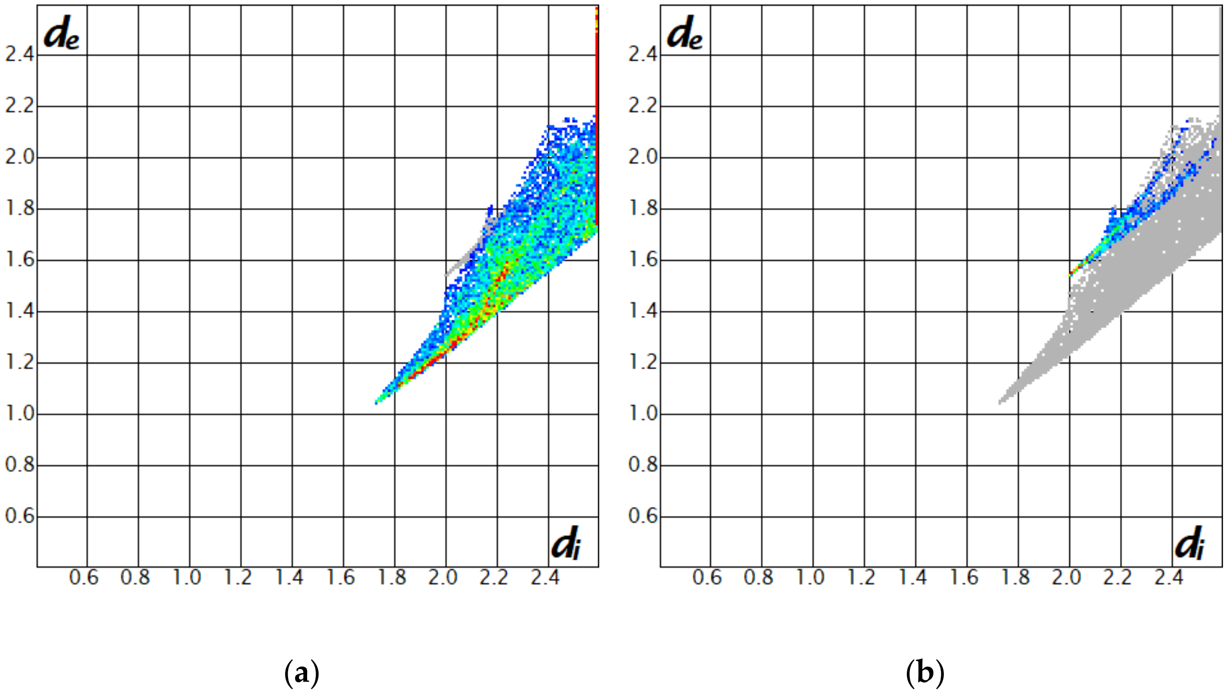


**Figure S14.** I…H (**a**) and I…O (**b**) contact fingerprints for the I5 atom in the crystal structure of **3-I**.

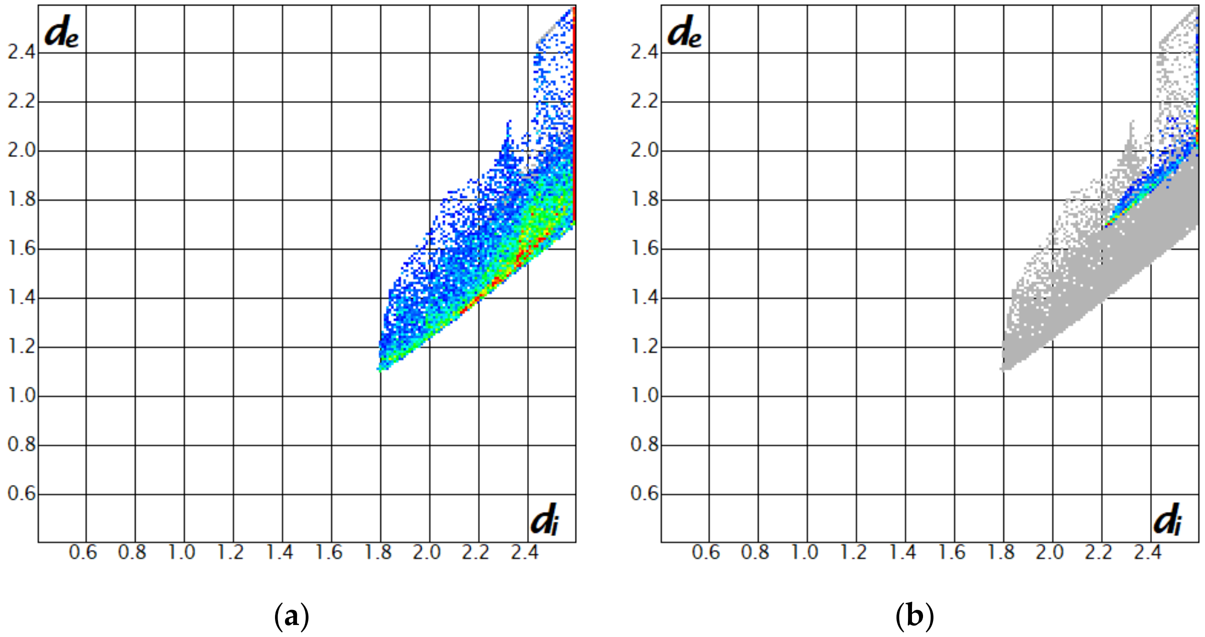


**Figure S15.** I…H (**a**) and I…O (**b**) contact fingerprints for the I6 atom in the crystal structure of **3-I**.

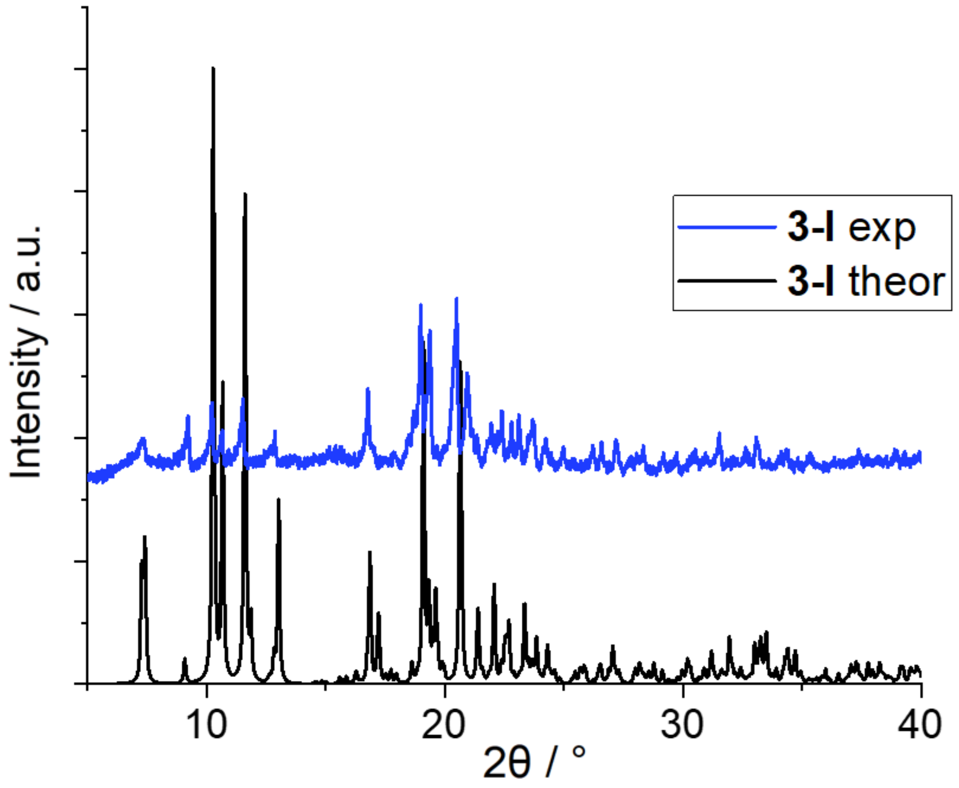


**Figure S16.** Experimental and theoretical PXRD patterns of **3-I**.

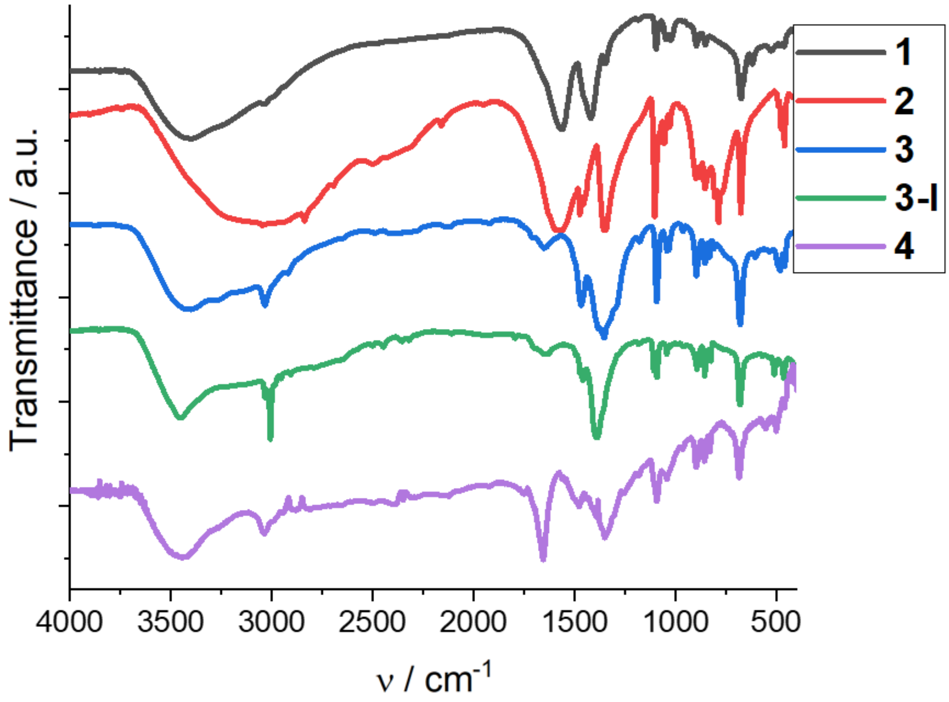


**Figure S17.** Infrared spectra of **1** – **4** and **3-I**.

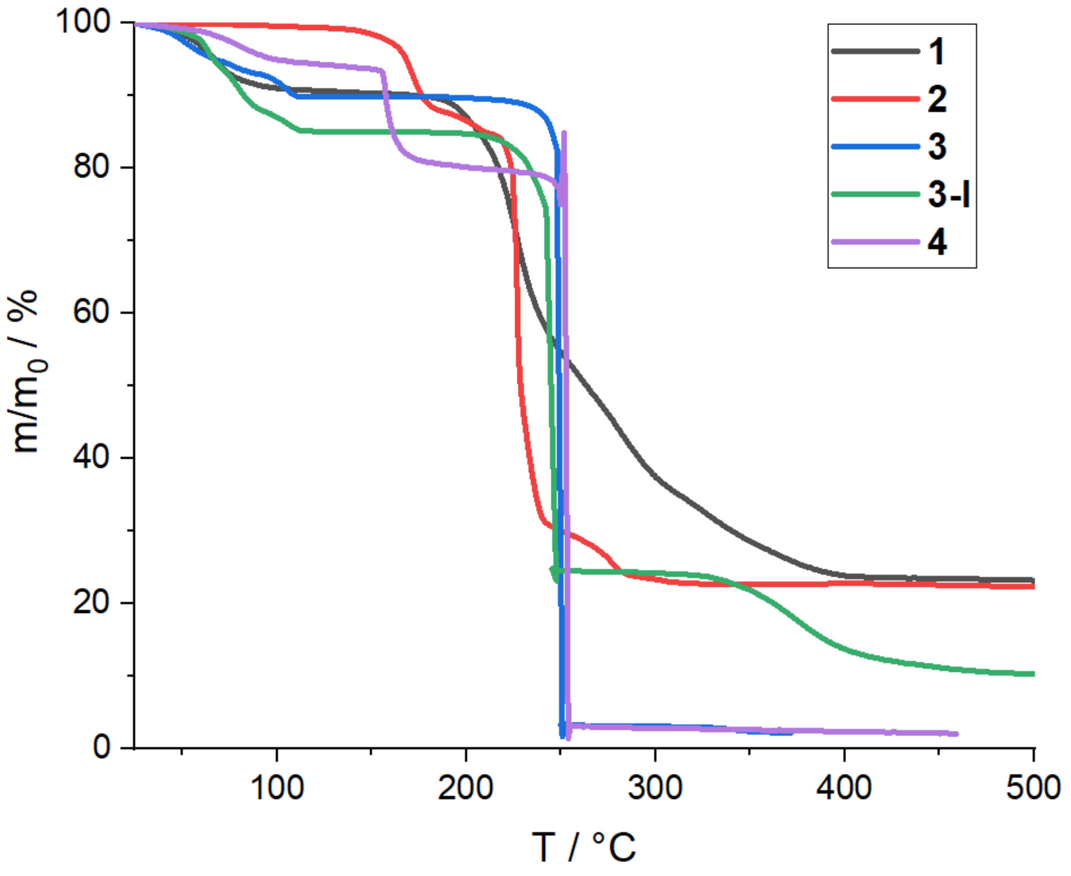


**Figure S18.** TG plots for **1 – 4** and **3-I**.
